# Characterization of Dextran Sodium Sulfate-Induced Inflammation and Colonic Tumorigenesis in *Smad3* ^−/−^ Mice with Dysregulated TGFβ

**DOI:** 10.1371/journal.pone.0079182

**Published:** 2013-11-11

**Authors:** Audrey Seamons, Piper M. Treuting, Thea Brabb, Lillian Maggio-Price

**Affiliations:** Department of Comparative Medicine, University of Washington, Seattle, Washington, United States of America; CWRU/UH Digestive Health Institute, United States of America

## Abstract

There are few mouse models that adequately mimic large bowel cancer in humans or the gastrointestinal inflammation which frequently precedes it. Dextran sodium sulphate (DSS)-induces colitis in many animal models and has been used in combination with the carcinogen azoxymethane (AOM) to induce cancer in mice. *Smad3* ^−/−^ mice are deficient in the transforming growth factor beta (TGFβ) signaling molecule, *SMAD3*, resulting in dysregulation of the cellular pathway most commonly affected in human colorectal cancer, and develop inflammation-associated colon cancer. Previous studies have shown a requirement for a bacterial trigger for the colitis and colon cancer phenotype in *Smad3^−/−^* mice. Studies presented here in *Smad3^−/−^* mice detail disease induction with DSS, without the use of AOM, and show a) *Smad3* ^−/−^ mice develop a spectrum of lesions ranging from acute and chronic colitis, crypt herniation, repair, dysplasia, adenomatous polyps, disseminated peritoneal adenomucinosis, adenocarcinoma, mucinous adenocarcinoma (MAC) and squamous metaplasia; b) the colon lesions have variable galactin-3 (Mac2) staining c) increased DSS concentration and duration of exposure leads to increased severity of colonic lesions; d) heterozygosity of *SMAD3* does not confer increased susceptibility to DSS-induced disease and e) disease is partially controlled by the presence of T and B cells as *Smad3* ^−/−^ *Rag2* ^−/−^ double knock out (DKO) mice develop a more severe disease phenotype. DSS-induced disease in *Smad3* ^−/−^ mice may be a useful animal model to study not only inflammation-driven MAC but other human diseases such as colitis cystica profunda (CCP) and pseudomyxomatous peritonei (PMP).

## Introduction

Inflammatory bowel disease (IBD) is characterized by chronic and relapsing inflammation of the gastrointestinal tract which is associated with an increased risk of developing colitis-associated cancer [1], [2]. There are a number of animal models, including those with inflammatory disease components, being used to investigate colon cancer [3], [4], [5] and these models recapitulate different aspects of colitis and associated cancer in humans [5]. Varied mechanisms influence the initiation and progression of tumors in these models including genetic susceptibility, inflammation, and use of carcinogens, however, not all aspects of the human disease are practical or convenient to study in any one model [5]. Further refinement and characterization of current animal models, as well as investigation of new models, is warranted in order to recapitulate the diverse aspects of the human disease [5], [6].

Altered TGFβ signaling has been associated with bowel inflammation as well as the development of colon cancer [3], [7], [8]. TGFβ signaling under normal conditions is important in maintaining intestinal homeostasis by skewing gut immune responses toward a tolerogenic state [3], [9], [10]. In the context of chronic bowel inflammation, alteration or loss of TGFβ-mediated immune regulation in combination with the effects of dysregulated TGFβ signaling in damaged epithelium and the resulting insufficient control of cell growth, can lead to tumor development [7]. The most common type of TGFβ signaling alteration in human colon cancer is via mutations in the TGFβ type II receptor, present in 30% of all colorectal cancers [8]. Additionally, mutations or loss of heterozygosity in the Smad2 or Smad4 genes, which are both involved in TGFβ signal transduction [11], are also present in human colon cancers [8]. In mice, deletion of TGFβRII in colonic epithelium leads to development of carcinoma in animals either treated with carcinogen or that are genetically susceptible to polyp formation via APCmin [12], [13]. Studies in compound mutant mice (*Apc^−/−^* and *Smad4^−/−^)* indicate that mutations in *Smad4*, which encodes a protein involved in TGFβ signaling, play a significant role in malignant progression of colorectal tumors [14]. Deficiency in another TGFβ signaling molecule, SMAD3, also leads to development of metastatic colorectal cancer [15].

We have previously demonstrated that development of colitis and colon cancer in *Smad3^−/−^* mice [16], [17] requires a trigger, such as infection with enterohepatic *Helicobacter*, to induce disease and others have shown that dextran sodium sulfate (DSS) serves as a chemical trigger to induce colon cancer in *Smad3^−/−^* mice [18]. DSS is a popular means to induce bowel damage and subsequent inflammation in a number of models [19]. Our studies reported here further characterize DSS-induced colitis and cancer in *Smad3* ^−/−^ mice. We provide details on the gross, histopathologic and immunohistochemical findings demonstrating a spectrum of lesions ranging from acute and chronic colitis, crypt herniation, repair, dysplasia, adenomatous polyps, disseminated peritoneal adenomucinosis, adenocarcinoma, mucinous adenocarcinoma (MAC) and squamous metaplasia; additionally some animals displayed features of CCP and PMP.

## Methods

### Ethics Statement

All animal studies were performed with strict adherence to the Guide for the Care and Use of Laboratory Animals of the National Institutes of Health. All animal procedures were approved by the University of Washington Animal Care and Use Committee under protocol #2436-12.

### Mice, Diets and Induction of Colitis

*Smad3* ^−/−^ (129S2/SvPasIco-*Madh3^tm1Par^*/J) mice [15] were originally obtained from the Jackson Laboratory (Bar Harbor, ME) and generated using heterozygous or homozygote breeding trios [16] at the University of Washington (UW). *Smad3/Rag*-*DKO* mice were generated by intercrossing *Smad3^−/−^* mice with *Rag2* ^−/−^ (129S6/SvEvTac-*Rag2tm1Fwa*) mice [17]. Animals were housed in static micro-isolator (Alternative Design, Siloam Springs, AR) or individually ventilated cages (Allentown, Allentown, NJ ) containing corncob bedding (Andersons, Maumee, OH) and nestlets. Mice were fed autoclaved rodent chow (Animal Specialties, Portland, OR), irradiated Picolab Rodent Diet 20 #5053 (Lab Diet, St. Louis, MO ) or AIN93M (LabDiet/TestDiet, St. Louis, MO). Study animals were placed on autoclaved, acidified (pH 2.4–2.8) water in water bottles. Animals were maintained in a specific pathogen free facility within a *Helicobacter spp*.-free room. Sentinel mice (IcrTac:ICR from Taconic Farms (Albany, NY)) were tested every 3–4 months and were free from multiple viruses as previously published [20] in addition to lymphocytic choriomeningitis virus, ectromelia virus, minute virus of mice and mouse norovirus. Also, yearly fecal colon samples were screened for *Citrobacter rodentium*, nonlactose fermenting *Escherichia coli*, *Salmonella spp*., *Klebsiella spp*., and *Clostridium spp*. (Phoenix Laboratories, Everett, WA). Mice were confirmed to be free of *Helicobacter spp*. by fecal PCR (primer sequences published in Burich et al. [21]).

For colitis induction via dextran sodium sulfate (DSS), DSS (36,000–50,000 MW, cat # 0216011090, MP Biomedicals, Solon, OH) was prepared as a stock solution in autoclaved distilled water which was then diluted into autoclaved, acidified water from the animal facility and placed into autoclaved water bottles. 3% DSS in drinking water was given for 7 days. 1.5% DSS was given either as a single exposure for 7 days, or in 2–9 cycles where each exposure was given for 3–7 days followed by 14 days of untreated water before the start of the next cycle. Negative control animals received untreated water for the duration of the experiment. Age of the animals at the initiation of studies varied. Animals were weighed weekly and were also monitored for other disease symptoms including diarrhea, blood in feces, rectal prolapse, dehydration, and decreased body condition. Animals were monitored daily during peak of disease. Animals were euthanized when they developed 20% body weight loss or signs of severe disease (rectal prolapse, bloody diarrhea, loss of body condition), and tissue samples collected.

### Histopathology and Immunohistochemisty

Animals were sacrificed by CO_2_-asphyxiation and cardiocentesis and necropsy performed. The gastrointestinal tract was isolated and the colon separated from the cecum. Samples of colon, cecum and occasionally mesenteric lymph nodes were placed into 10% neutral buffered formalin. The colon was prepared in a “Swiss roll” technique [22], routine-processed for paraffin embedding and stained with hematoxylin and eosin. Cecum and colon were scored by PMT blinded to experimental groups. IBD scores were based on the severity of mucosal loss, mucosal epithelial hyperplasia, degree of inflammation and extent of pathology as modified from Fort et al. [23] and are further described in **Table 1** and illustrated in the figures. Dysplasia, described in **Table 2**, was classified according previously published criteria [5], [6] with the conservative modification that only frankly invasive lesions into and beyond the *tunica muscularis* and into the serosa were classified as neoplasia [17]. This conservative schema was employed to exclude the possibility of including pseudoherniation in the invasive lesion count. Because invasive lesions were often multifocal and variable in development, they were size-weighted and summed across all portions of the large bowel (cecum, proximal, mid and distal colon). Sizing scores were set as follows: 1∶2–3 crypts not penetrating serosa; 2∶4+ crypts not penetrating serosa; 3: <10 crypts penetrating serosa; 4: >10 crypts penetrating serosa or subgrossly visible serosal mass. Distribution score was determined by adding the number of colon segments affected by high grade dysplasia (grade 3 or 4). The maximum possible distribution score is 4. Metaplastic squamous epithelial lesions in the distal colon and rectum were also evaluated and scored as in **Table 3**. Dysplastic change in the squamous metaplasia was scored on a 0–4 scale, however the maximal dysplasia score in this study was 2.

**Table 1:** Grading of DSS IBD.

Mucosal loss*	Hyperplasia*	Inflammation*	Extent*,**
0- none	0- none	0- none	0- none
1- mild <5%	1- mild	1- mild mucosa only	1- <5%
2- moderate 6–30%	2- moderate	2- moderate mucosa and submucosa	2- 6–30%
3- marked 31–60%	3- severe with aberrant crypts	3- severe with abscesses, erosions	3- 31–60%
4- severe >61%	4- severe with herniation	4- severe with obliteration of architectureand ulceration or transmural	4- >60%

* These are scored for each section of large bowel (cecum, proximal colon, mid colon and distal colon) and summed for total IBD score.

** Two extent scores are included in the IBD score. Extent 1 =  % of intestine affected in any manner; Extent 2 = % percent of intestine affected by the most severe score.

**Table 2:** Dysplasia and invasive neoplasia scoring schema.

Dysplasia score^a^	Invasive tumor size weight^b^
0- no dysplasia	1- few cells to 3 crypts penetrating the tunica muscularis
1- indefinite low grade due to active inflammation/ulcers	2- 4+ crypts within tunica muscularis
2- low grade (includes atypical hyperplasia and adenomas)	3- <10 crypts penetrating tunica muscularis
3- high grade (includes adenomas and non-invasive carcinomas)	4- >10 crypts penetrating serosa or visible serosal mass subgrossly
4- high grade with invasion^C^ beyond tunica muscularis (carcinoma)	

a dysplasia score is assigned 0–4 for each section of large bowel, summed dysplasia are the sum of the scores in the individual segments (cecum, proximal colon, mid colon, distal colon).

b number of invasive tumors is multiplied by size weight in each segment of the large bowel (cecum, proximal colon, mid colon, distal colon). Invasive tumor score is the sum of these values across the bowel).

c diagnostic criteria for hyperplasia, adenoma, carcinoma and to distinguish invasion vs. pseudoinvasion (herniation) after Boivin et al 2003.

**Table 3:** Distal squamous cell metaplasia score*.

Metaplasia (0–4)	Extent**	Dysplasia***
0- normal	0-none	0- no dysplasia
1- few glands with metaplasia yet no increased thickness of the mucosa	1- <5%	1- indefinite low grade due to active inflammation/ulcers
2- few glands or increased thickness to 2X normal	2- 6–30%	2- low grade
3- multifocal to coalesced, thickness 2–3X normal	3- 31–60%	3- high grade
4- >3X normal thickness and/or presence of squamous pearls	4- >60%	4- high grade with invasion (carcinoma)

* Squamous cell metaplasia score was generated by adding the metaplasia score and two extent scores.

** Extent is for the distal half of the distal colon where longitudinal mucosal folds start. Two extent scores are included in the total squamous metaplasia score. Extent 1 =  % of distal colon affected in any manner; Extent 2 = % percent of distal colon affected by the most severe score.

*** The highest grade dysplasia noted in the *Smad3* ^−/−^ studies was 2 (low grade) but the higher scores are included here for completion.

Immunohistochemistry (IHC) was performed at the University of Washington Histology and Imaging Core Research Laboratory. The following reagents and antibodies were used: rabbit anti-bovine cytokeratin (Cat # Z0622, Dako North America, Inc., Carpinteria, CA), rat anti-mouse Galectin-3 (Clone M3/38, Cat # CL8942AP, Cedarlane Laboratories USA Inc, Burlington, NC), rat anti-mouse F4/80 (Clone BM8, Cat # MF48000, Invitrogen, Grand Island, NY), unconjugated anti-rat IgG (H+L), mouse adsorbed, made in rabbit (Cat # AI-4001, Vector Laboratories, Burlingame, CA), Normal Rabbit IgG (Cat # AB-105-C, R&D Systems, Minneapolis, MN), Rat IgG2b isotype control (Cat # 553986, BD Biosciences, San Jose, CA), Hematoxylin Stain Solution, Harris Non-Mercuric (Cat # 12013, Newcomer Supply, Middletown, WI) and Bond Hematoxylin Counterstain. All staining procedures were run on a Leica Bond Automated Immunostainer (Leica Biosystems, Buffalo Grove, IL) with Leica Bond reagents. Antibodies were detected with Bond Polymer DAB Refine and Bond Mixed Refine (DAB) detection buffers. Slides were counterstained with a hematoxylin counterstain.

### Statistical Analysis

Survival curves were compared using Mantel-Cox Log Rank test in GraphPad prism (GraphPad Software Inc., La Jolla, CA). Animals that died prior to the endpoint that were not confirmed IBD-related deaths were censored. IBD scores, tumor scores, summed dysplasia and distribution scores of DSS-treated mice were compared using Mann-Whitney T test. Four comparisons were made, 1) 1.5% DSS *Smad3* ^−/−^ vs. 1.5% DSS *Smad3/Rag-DKO* 2) 1.5% *Smad3* ^−/−^ vs. DSS cycles *Smad3* ^−/−^3) DSS cycles *Smad3* ^−/−^ vs. DSS cycles *Smad3/Rag-DKO* and 4) 1.5% *Smad3/Rag-DKO* vs. DSS cycles *Smad3/Rag-DKO*. Wilcoxon signed rank test was used to determine if treatment groups were significantly different from zero. Data were tested for normality and equal variances using the D’Agostino and Pearson omnibus normality test and Bartlett’s test, respectively, using Graphpad Prism 5 software. Even with data transformation, the data did not satisfy the conditions for parametric or nonparametric ANOVA. Incidence of dysplasia and invasive tumors in treatment groups was compared by Fisher exact test. Statistical analysis was done using Graphpad Prism 5 or Excel 2007.

## Results

### *Smad3* ^−/−^ Mice are Highly Susceptible to DSS-induced Colitis and Colon Cancer compared with *Smad3* ^+/−^ Animals

**Figure 1:**
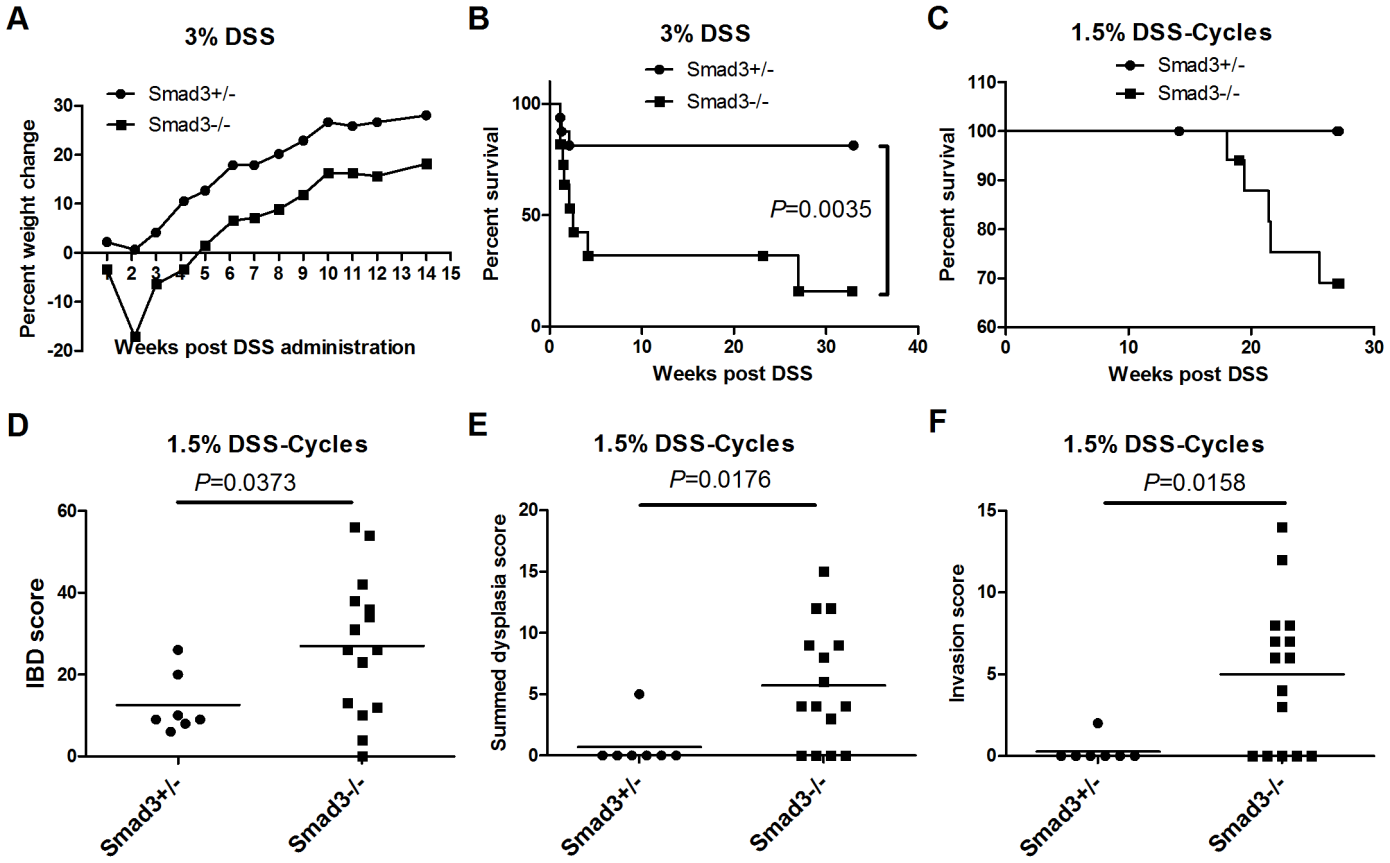
*Smad3^−/−^* mice are more susceptible to DSS-induced colitis and cancer than *Smad3^+/−^* mice. (A) Percent weight change of *Smad3^+/−^* and *Smad3^−/−^* mice treated with 3% DSS in drinking water for 7 days. Mice were euthanized when they reached endpoint criteria as outlined in Methods. week 1, n = 16 and n = 11; week2, n = 13 and n = 6; week3, n = 13 and n = 4; week 5 and after, n = 13 and n = 3 (*Smad3^+/−^* and *Smad3^−/−^*, respectively). (B) Survival analysis of mice treated with 3% DSS in (A). Endpoint 33 weeks. (C) Survival analysis (27 week endpoint) of *Smad3^+/−^* and *Smad3^−/−^* animals treated with 9 cycles of DSS. IBD score (D), Summed dysplasia score (E) and Invasion score (F) of same study as in (C). P-values (D–F) derived from a Mann-Whitney test.

*Smad3* ^−/−^ mice are exquisitely sensitive to damage by exposure to DSS. In initial experiments 3% DSS was given for 7 days. This treatment regimen was similar to one previously used in wild-type 129/SvPas mice (3.5% wt/vol DSS for 5 days) where no mortality occurred after treatment [24]. Deane et al. also used 2.5% DSS for 7 days treatment in *Smad3* ^−/−^ mice; however, this study did not discuss disease severity or mortality [18]. Using this regimen (3% DSS), *Smad3* ^−/−^ mice developed rapid, severe clinical disease characterized by weight loss, bloody diarrhea and dehydration. This differed significantly from our previously described induction of disease in this strain using *H. bilis,* in which there is rare mortality and colitis clinically resolves yet is followed by tumor development [16]. Signs of colitis also developed in *Smad3^+/−^* mice, however, on average, clinical signs such as weight loss and presence of bloody diarrhea were less severe compared to *Smad3* ^−/−^ mice. By one week post DSS administration, body weights of 7/11 *Smad3* ^−/−^ and 5/16 *Smad3^+/−^* mice had dropped below initial body weight resulting in an average weight change (**Figure 1A**) of minus 3.6% (n = 11) in *Smad3* ^−/−^ mice, compared to a 2.2% (n = 16) average weight gain in *Smad3^+/−^* animals receiving DSS treatment. By two weeks post DSS 3/16 *Smad3^+/−^* and 5/11 *Smad3* ^−/−^ mice had been euthanized. Body weights of 7/13 *Smad3^+/−^* and all of the remaining *Smad3* ^−/−^ mice had gone below initial starting weight. By three weeks, body weights of most surviving animals had started to recover (**Figure 1A**). Survival analysis demonstrated reduced survival in *Smad3* ^−/−^ mice (median survival 2.57 weeks post DSS administration, n = 11) compared to *Smad3^+/−^* mice (median survival was undefined as 13/16 mice survived to the study endpoint at 33 weeks, **Figure 1B**). Two *Smad3^−/−^* animals in this study lived long enough to develop invasive neoplasia; one tumor was found in an animal necropsied 4 weeks post DSS treatment and the other animal survived to 27 weeks and also developed tumors. In our colony, *Smad3^−/−^* mice (9 months and older) given untreated water and maintained *Helicobacter*-free do not develop colitis or colon cancer [16].

Although our results indicated that *Smad3* ^−/−^ mice were more susceptible to DSS colitis than *Smad3* ^+/−^ mice, the majority of DSS-treated *Smad3* ^−/−^ mice in this study did not survive long enough to develop dysplasia or colon tumors. Hence, we explored a reduction in DSS dosing, along with multiple rounds of treatment in *Smad3* ^−/−^ animals to characterize dysplasia and tumor development. Cyclical administration of 1.5% DSS resulted in less severe clinical disease and all DSS-treated mice (17 *Smad3* ^−/−^ and 7 *Smad3^+/−^*) lived to at least 18 weeks post initial DSS exposure. Mice were followed for a total of 27 weeks. Generally, mice gained weight and average percent weight gain was not statistically different between *Smad3* ^−/−^ and *Smad3* ^+/−^ mice (data not shown). Despite reduced dosing with DSS leading to decreased symptoms of acute disease and increased survival, care had to be taken to closely match feed and caging conditions between studies to ensure survival past the acute phase of disease. We found that both diet and caging conditions significantly impacted disease severity in response to treatment with 1.5% DSS (**Figure S1**), indicating that titration of DSS exposure may be required as housing conditions of animals are changed. Although most *Smad3^−/−^* mice survived to endpoint, disease signs consistent with tumor development (diarrhea, hunched posture, blood in feces, weight loss, palpable mass in abdomen) necessitated euthanasia of 5/17 *Smad3* ^−/−^ mice (between 19.4 to 25.6 weeks) prior to the study endpoint of 27 weeks (**Figure 1C**).

### DSS Induced IBD, Dysplasia and Colonic Tumors in *Smad3* ^−/−^ Mice and Mild to Moderate Disease in *Smad3^+/−^* Mice

To characterize the histopathological changes associated with DSS-induced disease in *Smad3* ^−/−^ mice and *Smad3^+/−^* mice, large bowel from each animal were scored for IBD, dysplasia and invasive neoplasia. Endpoint IBD scores were significantly increased in *Smad3* ^−/−^ mice compared to *Smad3^+/−^* mice (mean IBD score = 27 vs. 12.6, respectively, *P* = 0.0373, **Figure 1D**). Summed dysplasia scores (described in **Table 2**) are shown in **Figure 1E**. Dysplasia scores in *Smad3* ^−/−^ mice correlated with IBD scores (Spearman’s r = 0.8904, *P*<0.001) and were significantly higher (*P* = 0.0176) than dysplasia scores in Smad3^+/−^ mice (mean dysplasia score = 5.7 vs. 0.71). Likewise, invasion scores (**Figure 1F**) also correlated with IBD scores (Spearman’s r = 0.7921, *P* = 0.0004) and were significantly increased in *Smad3* ^−/−^ versus Smad3^+/−^ animals (*P* = 0.0158). Invasive carcinoma was detected in only 1/7 (∼14%, proximal colon mucinous adenocarcinoma) *Smad3* ^+/−^ mice necropsied at the experimental endpoint (27 weeks) compared to *Smad3^−/−^* mice, where 10/15 animals (∼67%) developed invasive carcinoma (*P* = 0.0635). DSS-induced a wide spectrum of large bowel disease in *Smad3* ^−/−^ mice as described further below.

### Repeated DSS Exposure as well as Lack of T and B cells Increases Disease Severity in *Smad3* ^−/−^ Mice

**Figure 2:**
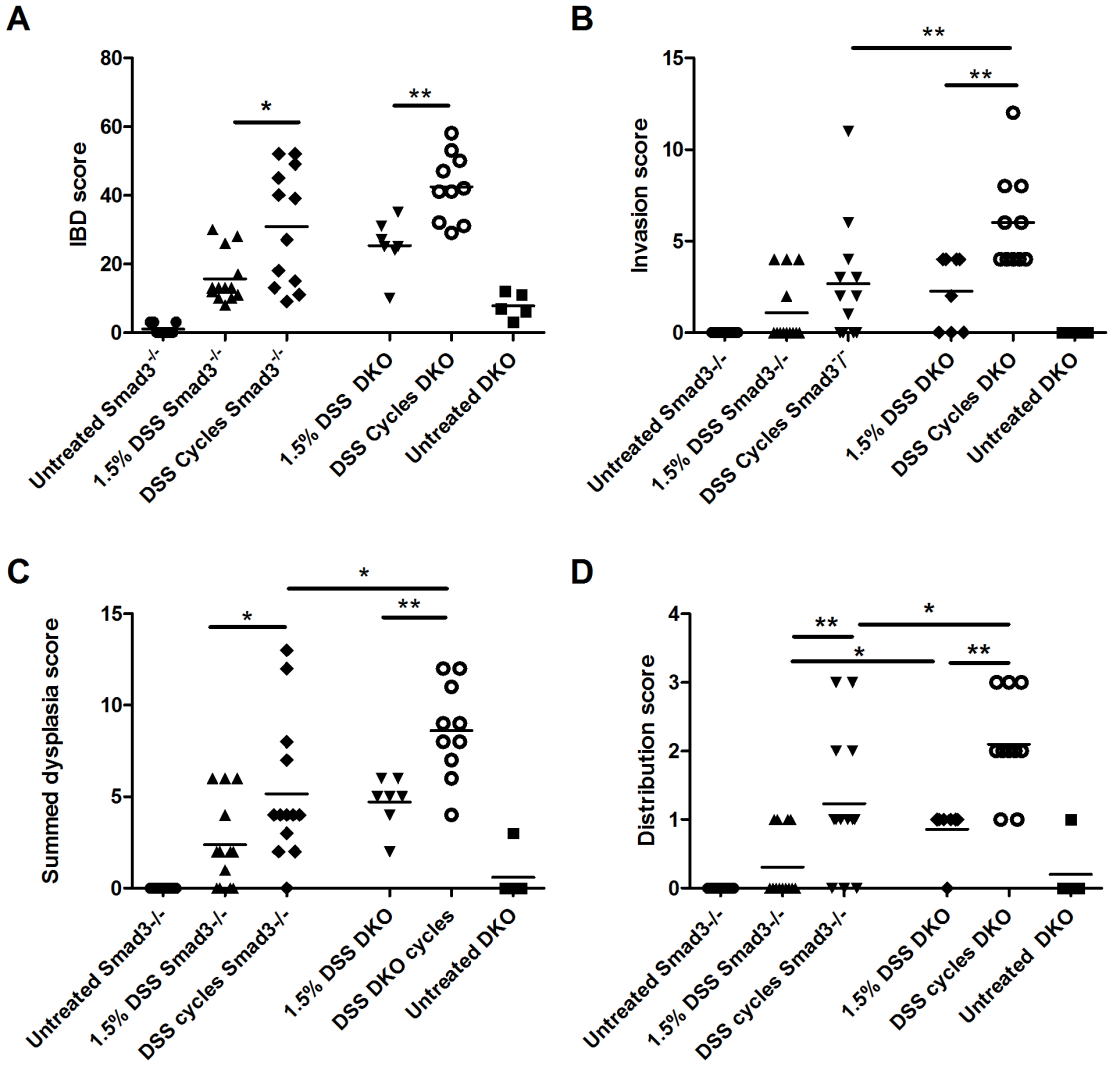
Histopathology scores of DSS-treated *Smad3^−/−^* and *Smad3/Rag-DKO* mice treated with different doses of DSS. *Smad3^+/−^*, *Smad3^−/−^* and *Smad3/Rag-DKO* (DKO) mice were treated with either a single DSS cycle or 9 cycles of DSS. Experimental endpoint was 17 weeks. A) IBD score, B) Invasion score, C) Summed dysplasia score and (D) Distribution score (as described in materials and methods) are shown for individuals in each treatment group. Negative control groups were all statistically different (significance not shown in figure) from their respective DSS-treated group except for untreated water vs. 1.5% DSS *Smad3^−/−^* in (B). *P≤0.05, **P≤*0.01.*

In order to investigate the role of T and B cells in inflammation and tumor development in this model, we compared DSS-induced disease in *Smad3* ^−/−^ and *Smad3/Rag-DKO* mice using two different DSS regimens (single cycle of 1.5% DSS for 7 days or 9 cycles of 1.5% DSS). A comparison of scores between *Smad3* ^−/−^ and *Smad3/Rag-DKO* mice are shown in **Figure 2**. Exposure to DSS cycles resulted in significantly higher IBD scores in both *Smad3* ^−/−^ and *Smad3/Rag-DKO* mice when compared to single DSS exposures (**Figure 2A** **)**. IBD scores were not significantly different between DSS-treated *Smad3* ^−/−^ and *Smad3/Rag-DKO* mice (**Figure 2a**), although increased invasion scores, dysplasia scores and distribution of high grade dysplasia were seen in *Smad3/Rag-DKO* when compared to *Smad3^−/−^* (**Figure 2A–D**). Generally, repeated exposure to DSS (cycles) was associated with higher IBD scores (**Figure 2A**), invasion scores (**Figure 2B**), dysplasia scores (**Figure 2C**) and increased distribution of high grade dysplasia throughout the colon (**Figure 2D**).

### Heterogygosity of *Smad3* does not Confer Susceptibility to DSS-induced Colitis or Tumors

In order to determine if heterozygosity for *SMAD3* confers susceptibility of mice to DSS-induced colitis and tumors, *Smad3* ^+/−^ and WT mice were also included in the study shown in **Figure 2** and were exposed to 1.5%, 3% and repeated DSS cycles **(Figure S2).** Severity of DSS-induced IBD was not significantly different between *Smad3* ^+/−^ and WT mice regardless of DSS concentration and single or repeated administration (**Figure S2A**). Additionally, no invasive neoplasia was detected in *Smad3* ^+/−^ or WT mice at 17 weeks post DSS exposure. However, low grade dysplasia developed in the distal colon of 1/6 and 2/12 3%-DSS-treated *Smad3* ^+/−^ and WT mice, respectively, at the 17-week end point and 1/15 DSS-cycles treated *Smad3* ^+/−^ developed high grade dysplasia (grade 3) in the cecum (**Figure S2B**).

### Necropsy Findings and Histopathology of DSS-induced Colitis and Tumors in *Smad3* ^−/−^, *Smad3^+/−^*, and *Smad3/Rag-DKO* Mice

**Figure 3:**
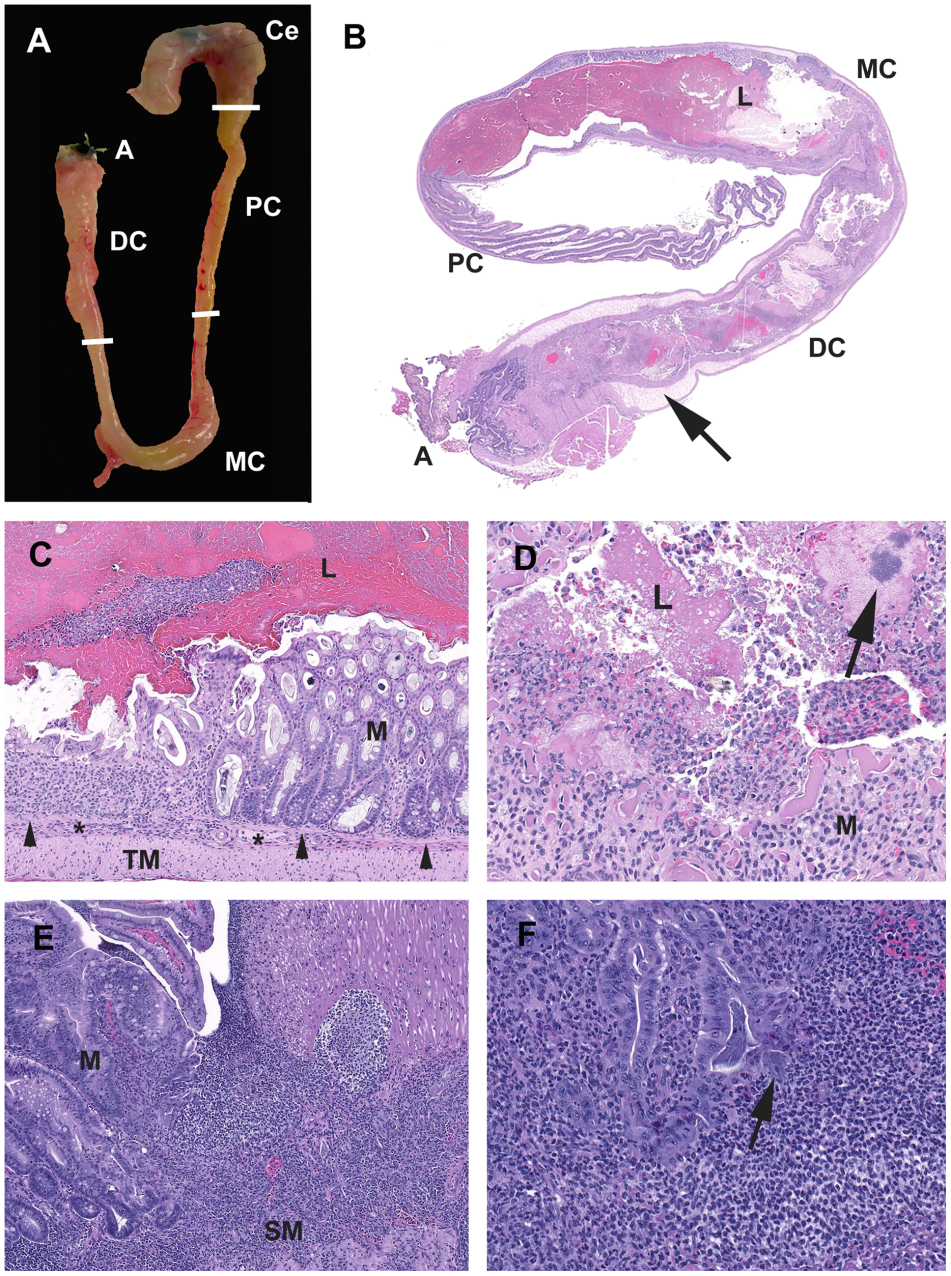
Characteristics of acute typhlocolitis induced by DSS. (A) *Smad3^−/−^*1.5% DSS. Cecum (**Ce**) and colon (**PC**, proximal; **MC**, mid; **DC**, distal; **A**, anus). The cecum is contracted with darkly discolored contents. The colon lacks formed fecal pellets. The mid and distal colon are thickened and turgid. (B) *Smad3^+/−^*3% DSS. Subgross hematoxylin and eosin-stained section of DSS-exposed colon. The cecum has been removed. The mid and distal colon the luminal (**L**) contents are fluid with no formed fecal pellets. The submucosa (arrow) is markedly expanded by edema. (C) *Smad3^+/−^*3% DSS. Mid colon at the junction of erosive mucosal loss and effacement by inflammatory cells adjacent to intact mucosa (M). Note the muscularis mucosae (arrowheads) and the expansion of the submucosa (*) with inflammatory cells. *Tunica muscularis* as indicted (**TM**). (D) *Smad3^+/−^*3% DSS. Exudate adherent to ulcerated mucosa contains degenerate inflammatory cells, erythrocytes, fibrin and proteinaceous fluid with hazy coccoid bacterial colonies (arrow). (E) *Smad3^−/−^*3%DSS. Chronic-active proliferative and ulcerative colitis. The mucosa adjacent to an ulcer is proliferative with irregular and angular glands with loss of goblet cells, and increased mitotic figures. (F) *Smad3^−/−^*1.5% DSS. Glands adjacent to areas of active inflammation are classified as indefinite for dysplasia due to active inflammatory milieu. Dilated crypt filled with filamentous bacteria with a portion escaping into the adjacent lamina propria/submucosa (arrow).

**Figure 4:**
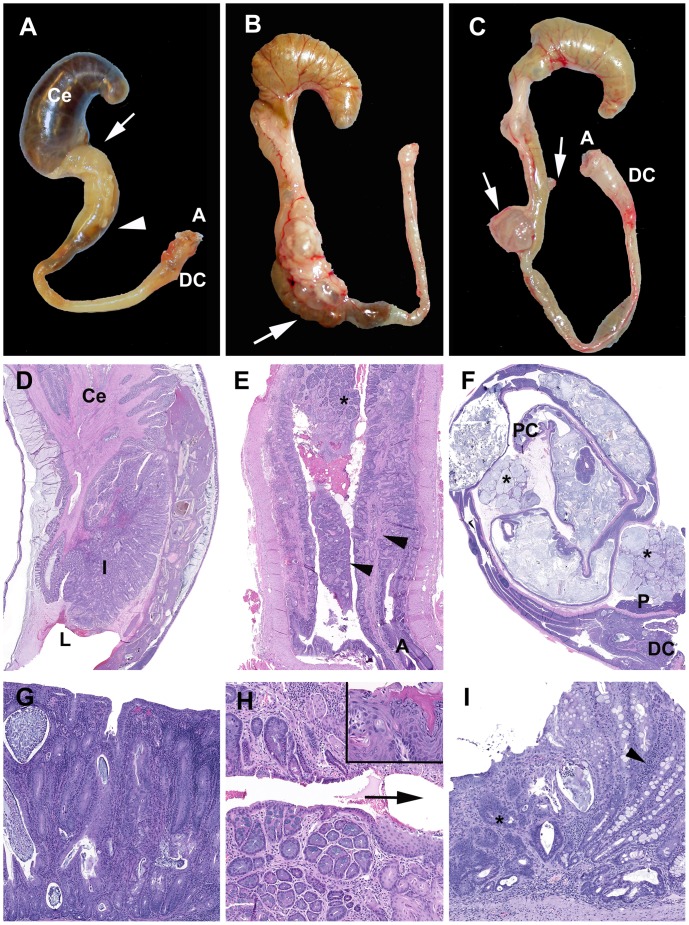
Chronic typhlocolitis and associated secondary lesions induced by DSS. (A–C) Examples of chronic lesions. (A) *Smad3^−/−^* DSS cycles. Cecum (Ce) and colon (PC, proximal; MC, mid; DC, distal; A, anus). The arrow and arrow head delineate region presented histologically in (D). (B) *Smad3/Rag-DKO* 1.5% DSS. A large multicystic mass is accompanied by retention of fecal pellets (arrow) consistent with partial obstruction. (C) *Smad3^−/−^* DSS cycles. Multifocal mucin-filled cystic masses (arrows) are present. Ill-formed fecal pellet is present in the mid colon and the distal colon is thickened and opaque. (D–F) Subgross histological sections. (D) Proximal colon see (A). The gross appearance is due to an intussception (**I**) into the dilated distal lumen (**L**). (E) *Smad3^+/−^*3% DSS. Distal colon and anus (**A**). The longitudinal mucosal folds of the distal colon (arrowheads) and the transition from glandular to metaplastic mucosa (asterisk) are indicated. (F) *Smad3^−/−^* DSS cycles. Multifocal mucinous serosal and mesenteric masses (asterisks). The distal colon (**DC**) and pancreas (**P**) are indicated. (G) Chronic severe proliferative and lymphohistiocytic colitis with cryptitis and crypt abscesses. (H) Higher magnification of asterisked region in (E). Inset: Squamous regions may have areas of prominent cornification (right side) and non-cornified areas (left side). Arrow indicates direction of the anus. (I) *Smad3^−/−^*3%DSS.The spectrum of chronic mucosal lesions includes moderate proliferative lymphohistiocytic colitis (arrow head) and atypical glands (*) that are not associated with active inflammation or ulceration.

Acute changes induced by DSS in the *Smad3* ^−/−^ and Smad3^+/−^ mice are consistent with those previously reported in other DSS models [19], [25], [26]. Acute colitis grossly presented with turgid and thickened colons often with contracted ceca and devoid of formed fecal pellets (**Figure 3A**). Histologically acute lesions included erosive to necroulcerative typhlocolitis with variable intralumenal hemorrhages, fibrin and cellular debris (**Figure 3 B–D**). In the subacute phase, epithelial proliferation was present and often associated with active areas of ulceration (**Figure 3E–F**). Over time, the mice developed chronic typhlocolitis with secondary lesions such as intussusceptions, obstruction and serosal masses, however, this was rarely seen in *Smad3^+/−^* animals (**Figure 4 A–C**). Chronic lesions included mucosal proliferation, dense intramucosal inflammatory infiltrates, epithelial dysplasia, and distal colon squamous metaplasia **(** **Figure 4 D–I**).

**Figure 5:**
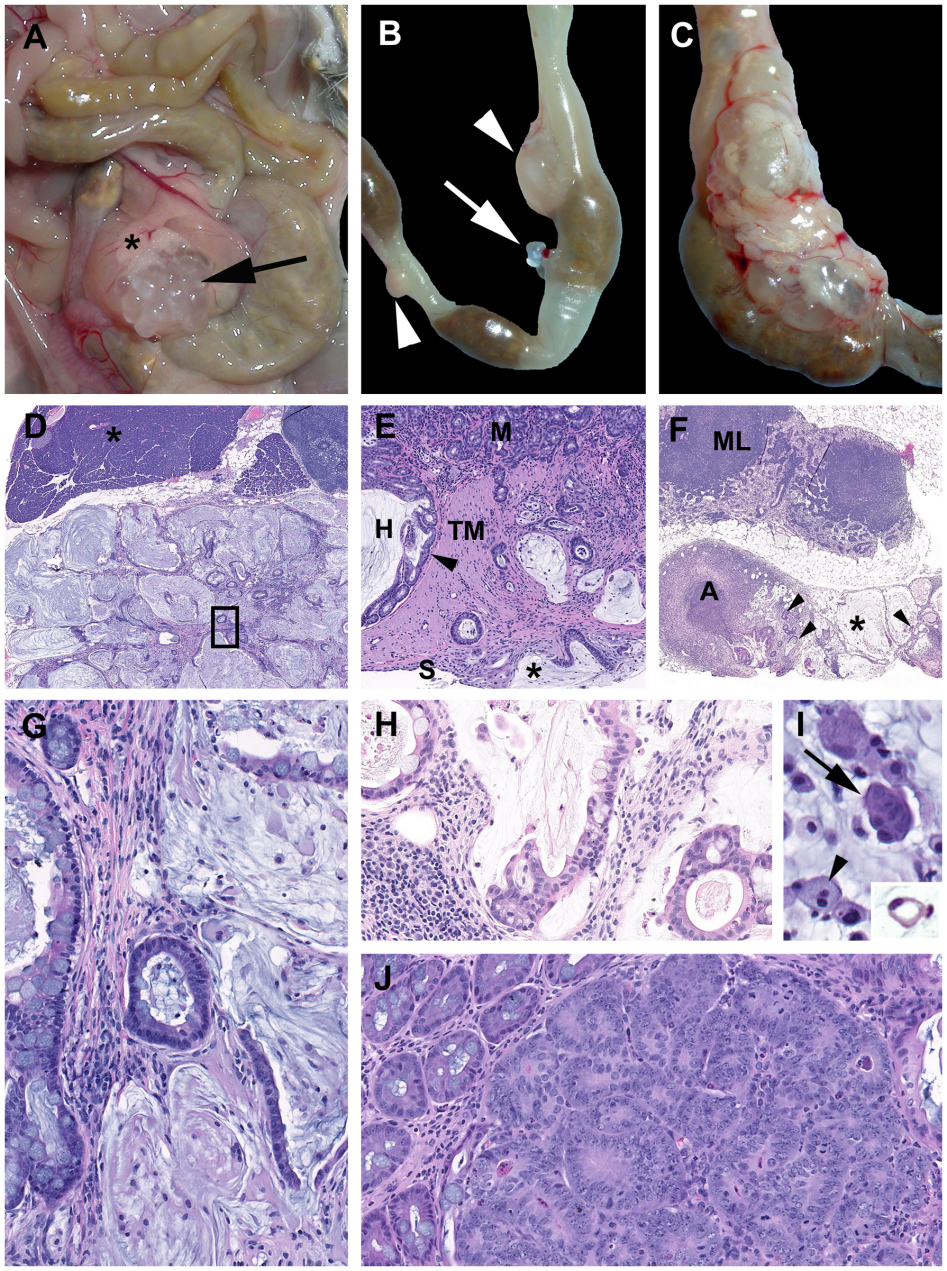
Mucinous neoplasia and high grade dysplasia induced by DSS. (A) *Smad3/Rag-DKO* DSS cycles. A cecal-colic multicystic mass (arrow) is present adjacent to the pancreas (asterisk). (B) *Smad3/Rag-DKO* 1.5%DSS. Multiple serosal masses are indicated on the mid to distal colon. (C) *Smad^−/−^* DSS cycles. A large multicystic mesenteric mass. (D) *Smad^−/−^* DSS cycles. Expansile mass in (A). Pancreas (asterisk) and mesenteric lymph node (upper right). Box region presented in (G). (E) Herniated proliferative mucosa (**H**) with compression the tunica muscularis (**TM**) with preservation of the submucosa (arrowhead). At left are invasive angular glands, dissecting mucin in the TM with focal penetration of the serosa (**S**) and intraperitoneal mucus (asterisk). (F) *Smad^−/−^* DSS cycles. Mesenteric implant of mucinous cysts (asterisk) with mucin-producing epithelial lined glands (arrowheads). Note abscess (**A**) and mesenteric lymph node (**ML**). (G) Boxed region in (D). Note dissecting lakes of mucin and isolated epithelium and epithelial rafts within the cysts. (H) Higher magnification of asterisked region in (F). (I) *Smad3^+/−^*3%DSS. Within mesenteric cysts are free large round cells (arrowhead) and clumps of basophilic cells (arrow) and rare signet rings (inset, Smad3−/− DSS cycles) (J) Foci of high grade dysplasia with in a hyperplastic polyp.

Neoplastic lesions noted in these studies are similar to those we previously reported with *H. bilis* infection [16], [17]. While neoplasia induced by *H. bilis* infection are primarily located in the proximal cecocolic junction [16], the preferred niche for *Helicobacter,* DSS-induced tumors occurred in multiple locations, including the proximal, mid and distal colon (**Figure 5 A–C**). Grossly, the larger masses were characterized as either multilocular straw-colored gelatinous masses with a grape cluster-like appearance (**Figure 5A**) or unilocular cream-colored and firm masses (**Figure 5B and C**) protruding into the serosa and expanding the attached mesentery. Histologically, opaque masses were often inflamed with numerous macrophages and variable neutrophils, whereas clear cysts were characterized as mucin-filled cysts lined by normal to mildly dysplastic epithelium and no associated inflammation. Most well-developed neoplasias were MAC (**Figure 5 A–I**); rarely, solid adenocarcinomas were also noted (**Figure 5 J**). The characteristic mucinous adenocarcinomas were composed of multiple mucin-filled cysts, lined by neoplastic epithelial cells and within mesenteric cysts were numerous large round cells with abundant foamy cytoplasm. Cysts were present within and penetrating the colonic muscular tunics and serosa. Often there was marked expansion into and compression of the mesentery and peritoneal structures including lymph nodes and occasionally the pancreas (**Figure 5D**). Free and dissecting mucus, mucin pools with isolated floating cells and a desmoplastic response were frequent **(** **Figure 5 G and H**). The epithelium lining the peritoneal cysts varied from well-differentiated to mildly dysplastic colonic epithelium with abundant mucus-producing cells and rare signet rings (**Figure 5 G–I**). Infrequently, free peritoneal mucous or epithelial lined mucus-filled cysts were noted in an individual animal with no evidence of a primary focus in the tissues examined histologically.

### Patterns of Galactin-3 Staining

**Figure 6:**
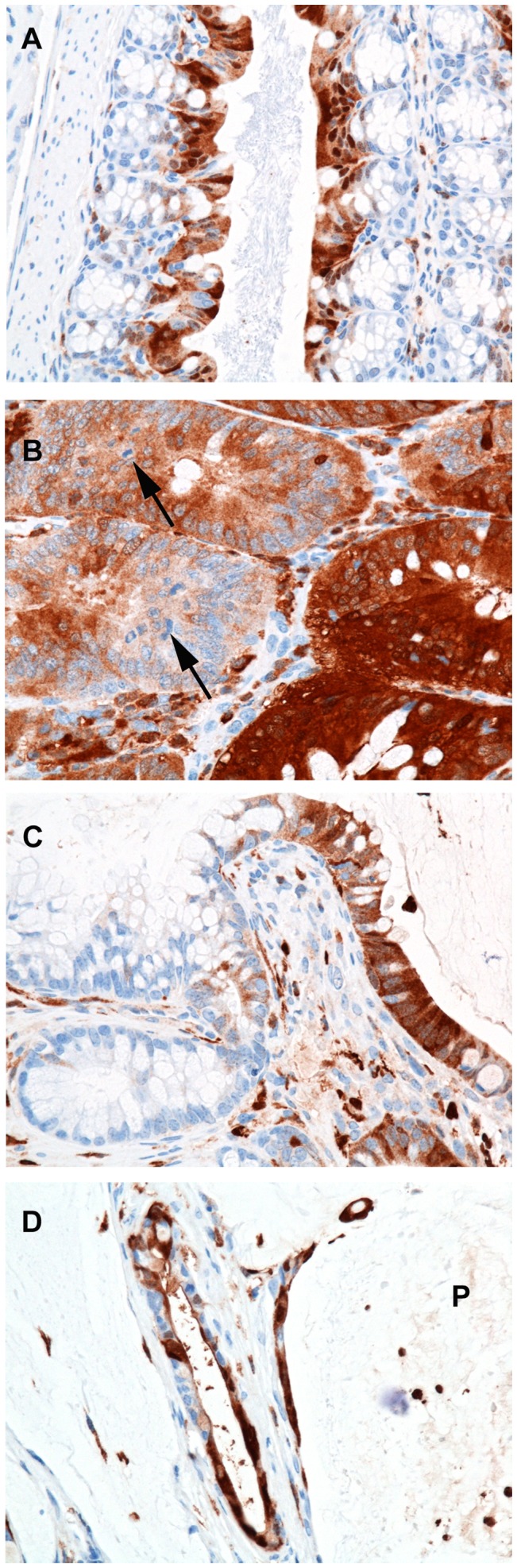
Patterns of galectin-3 expression. *Smad3^−/−^*3% DSS (A–C). Sections of formalin-fixed paraffin-embedded colon from a mouse with regions of normal proximal colon (A) mucosal adenomatous hyperplasia (B) and mucinous adenocarcinoma (C and D) immunohistologically stained for galectin-3. (A) In normal proximal colonic mucosa, galectin-3 expression is restricted to well-differentiated apical colonocytes with strong nuclear and lesser cytoplasmic signal. (B) Within adenomas there is loss of signal in the proliferative cells (arrows, mitotic figures) and staining cytoplasmic signal with indistinct to absent nuclear signal. (C) Within invasive crypts deep within the tunica muscularis (see A) there is loss of signal with gradual increase in signal as cells migrate into the peritoneal cavity and presumably differentiate to a mucinous phenotype. (D) *Smad3/Rag-DKO*, 1.5% DSS Strong cytoplasmic signal is present in the neoplastic epithelium lining mucinous peritoneal cysts and in free floating cells within the mucin pools (**P**).

**Figure 7:**
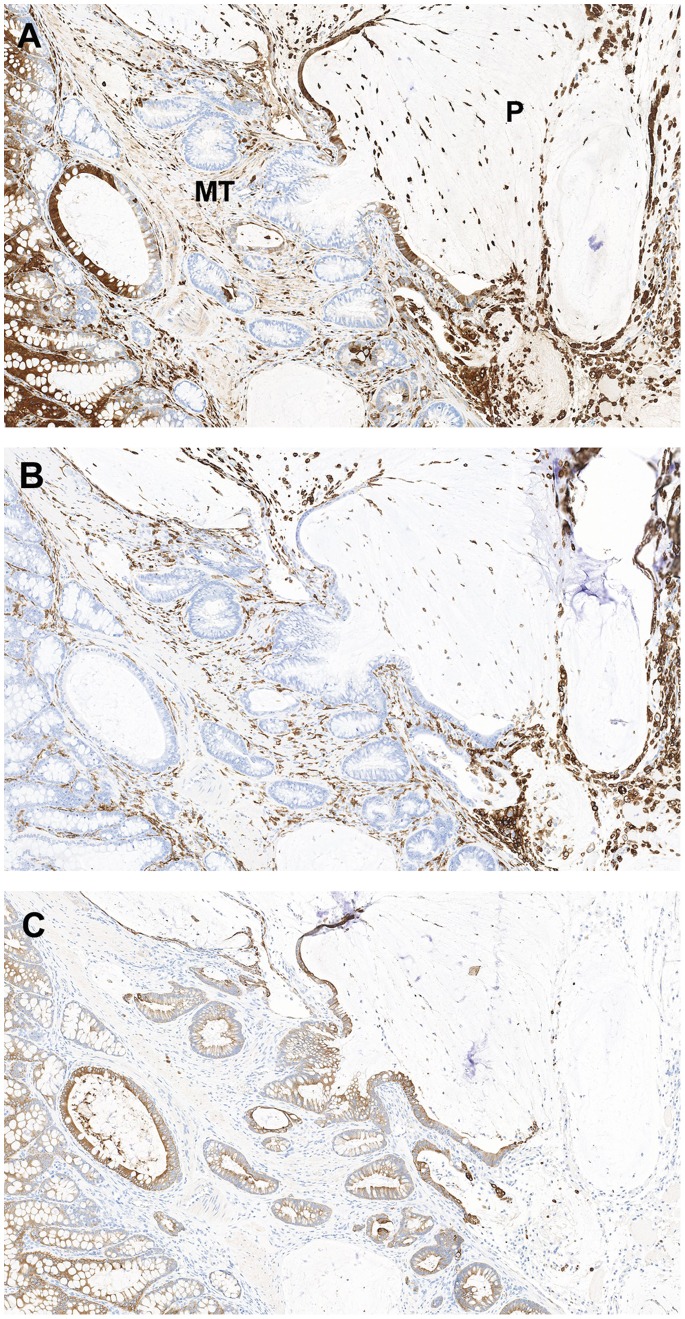
Differential staining patterns between macrophage and epithelial cell markers. Representative images of formalin-fixed paraffin-embedded mucinous adenocarcinoma from a *Smad3/Rag-DKO* 1.5% DSS animal stained for galectin-3 (A), the macrophage marker F4/80 (B) and wide spectrum cytokeratin (C). (A) Galectin-3 staining is variable in the neoplastic epithelium with loss of signal in the invasive and less differentiated glands within the muscular tunics (MT). There is increased cytoplasmic signal in the epithelium lining the peritoneal mucinous lesions (P indicates peritoneal cavity). Note that activated macrophages express galectin-3 and F4/80 (B), whereas only the colonic epithelium is positive for cytokeratins (C).

Galactin-3 (gal-3; MAC2) is a β-galactoside-binding lectin present in the nucleus and cytoplasm; it has been linked to the behavior of colon cancer cells with down-regulation noted with tumor invasion and progression [27], [28], [29]. In contrast, others noted increased gal-3 staining with progression [30], [31]. To further characterize the mucinous lesions in DSS-treated mice, we examined expression patterns and signal intensity of gal-3 in normal, hyperplastic and neoplastic lesions from three DSS-treated *Smad3^−/−^* and one *Smad3/Rag-DKO* mice. IHC staining for gal-3 demonstrated a spectrum of signal location in stained tissues (nuclear, cytoplasmic, and both nuclear and cytoplasmic) and intensity (light to intense). Staining patterns appeared similar between lesions in *Smad3^−/−^* and *Smad3/Rag-DKO*. There was intense nuclear and lesser cytoplasmic signal in normal well-differentiated colonocytes **(** **Figure 6A** **)**. With the development of proliferative disease, there was a relative loss of nuclear signal in adenomas to total loss of signal in the invasive crypts deep within the *tunica muscularis* **(** **Figure 6B–C** **)**. Increased staining was noted in cells within the peritoneal cavity and strong cytoplasmic signal was present in the neoplastic epithelium lining mucinous peritoneal cysts and in free-floating cells within the mucin pools (**Figure 6C–D** **and** **7A**). No differential gal-3 staining was noted between untreated *Smad3^−/−^* (n = 2) or untreated WT (n = 2) mice (data not shown). Because both macrophages and colonic epithelium express gal-3 [32], the macrophage specific marker, F4/80, and a wide-spectrum cytokeratin antibody were used to differentiate macrophages from epithelial cells in the mucinous lesions (**Figure 7 B–C**). The free floating cells were generally macrophages with fewer morphologically viable cytokeratin positive cells noted in the sections examined.

### DSS-induced Squamous Metaplasia and Low Grade Dysplasia in Distal Colon of DSS-exposed Mice

As has been noted with DSS colitis previously [19], [33], [34], the distal portion of DSS-treated colons often develop squamous metaplasia in concert with robust re-epithelialization. In both *Smad3^−/−^* and *Smad3^+/−^* mice, distal colons often appeared thickened and bulbous (**Figure 4A & C**). Histologically, the squamous metaplasia was characterized by distal colonic crypts lined by squamous epithelium (**Figure 4H**). The distal colonic hyperplastic and metaplastic lesions had variable galectin-3 staining with regions of nuclear and cytoplasmic, nuclear only or no signal within the proliferative regions (data not shown). Given the longitudinal mucosal folds present in the distal colon of mice and high potential for rectal prolapse in colitis, the distal colonic and rectal squamous lesions were conservatively classified by morphology (**Table 3**). There were no frankly invasive squamous lesions noted beyond the submucosa in numerous serial recuts of suspicious foci.

**Figure 8:**
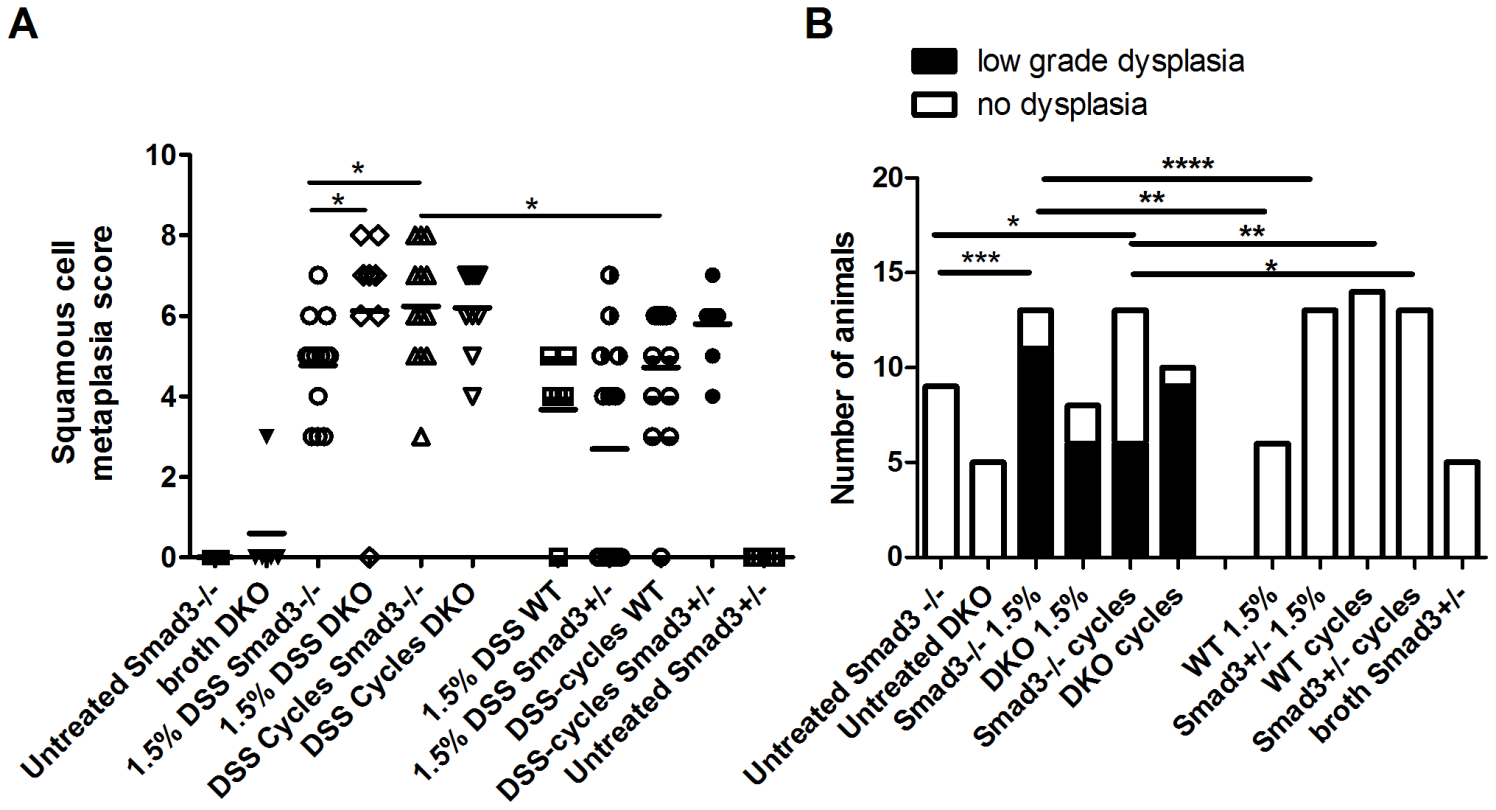
Squamous metaplasia and dysplasia in distal colons of DSS-treated *Smad3^−/−^* mice. *Smad3^+/−^*, *Smad3^−/−^* and WT mice were treated with either 1.5% DSS for one or 9 cycles. Experimental endpoint was 17 weeks. (A) Squamous cell metaplasia was scored as described in Table 3. Pairwise comparisons between DSS-treated groups were via Mann-Whitney test. B) The same region of the distal colon as in (A) was scored for squamous cell dysplasia. No high grade (grade 3 or 4) dysplasia was detected. Incidence of dysplasia was compared via Fisher exact test. *P≤0.05, **P≤0.01, ***P≤0.001, ****P≤*0.0001*.

Squamous cell metaplasia developed to varying degrees in all mice exposed to DSS regardless of the presence or absence of the *SMAD3* allele and a comparison in *Smad3^−/−^*, *Smad3^+/−^* and WT mice exposed to two different DSS regimens (single cycle of 1.5% DSS or 9 cycles of 1.5% DSS) is shown in **Figure 8A**. Increased exposure to DSS (1.5% DSS vs. DSS cycles) was associated with increased squamous metaplasia in both *Smad3* ^−/−^ and *Smad3* ^+/−^ mice (*P* = 0.0131 and 0.0039, respectively, Mann-Whitney). Additionally, *Smad3^−/−^* mice given DSS cycles had elevated squamous metaplasia scores compared to WT mice given the same treatment. No squamous metaplasia was detected in either *Smad3^−/−^* or *Smad3^+/−^* control animals given untreated water (**Figure 8A**). Although the occurrence of squamous metaplasia in the distal colon was not dependent on the absence of *SMAD3*, low grade dysplasia of squamous lesions only occurred in *Smad3^−/−^* mice (**Figure 8B**). Incidence of low grade dysplasia in either 1.5% DSS or cycles DSS-treated *Smad3* ^−/−^ mice was significantly higher (*P* = 0.0002 and 0.0461, respectively, Fisher exact test) than *Smad3* ^−/−^ animals given untreated water. Low grade dysplasia was not detected in any DSS-treated WT or *Smad3^+/−^* mice (**Figure 8B**).

## Discussion

DSS-induced colitis is a popular model used to study bowel inflammation because of the ease of administration and the ability to induce disease in many different strains of mice [24]. It is commonly used in concert with a carcinogen to induce inflammation-associated carcinogenesis [35]. We have utilized DSS to induce inflammation and cancer without the use of an additional carcinogen, in TGFβ signaling-deficient *Smad3^−/−^* mice [15], which are defective in one of the most common signaling pathways mutated in human colorectal cancer. Invasive carcinoma readily develops in DSS-treated *Smad3^−/−^* mice within a similar time frame seen in AOM/DSS-induced cancer models [35], [36], [37] and in a relatively short time frame compared to inflammation-induced cancer induced by DSS alone [36]. Also similar to AOM/DSS-induced cancer models, disease severity in DSS-treated *Smad3^−/−^* mice is modulated by altering the exposure of mice to DSS [38]. The spectrum of lesions that develop in DSS-treated *Smad3* ^−/−^ mice also exhibit characteristics of some human diseases such as colitis cystica profunda (CCP) and pseudomyxomatous peritonei (PMP), the latter of which has not been discussed in relation to other DSS-associated cancer models. Herein, we have described both the clinical course as well as the spectrum of pathology induced by DSS in *Smad3* ^−/−^ mice.

Acute DSS-colitis is thought to be initiated by the toxic effects of DSS on the gut epithelium [26], [39], with crypt loss and erosions being the first histopathological changes prior to inflammation. Although acute clinical disease peaks day 7–9 post administration of DSS in WT mice, tissue repair is evident at this stage. Significant tissue repair restoring the damaged epithelium typically occurs by two weeks post DSS. By five weeks, most of the epithelium has regenerated except in foci which have developed chronic colitis [40]. Our studies demonstrate that SMAD3 is important in the repair phase of DSS-induced colitis, as most 3% DSS-exposed *Smad3^−/−^* mice succumbed to disease prior to 5 weeks post DSS treatment. How deficiency of SMAD3 affects tissue healing has been an active area of research with conflicting results depending on the tissue studied and model used [41], [42], [43], [44]. In the gut, increased proliferation of the mucosal epithelium in response to injury has been shown in *Smad3^+/−^* mice [44] as well as *Smad3^−/−^* mice [41]. In the later study, using an acid injury approach, Owen et al. demonstrated increased proliferation of epithelial cells, no increased apoptosis, but decreased tissue repair. The authors show data to support the hypothesis that impaired healing of damaged intestinal mucosa in *Smad3* ^−/−^ mice was the result of disrupted cell migration to the wound [41]. Additional studies compliment these findings by demonstrating that monocyte infiltration of wounds is deficient in *Smad3^−/−^* mice [42] and that the lack of SMAD3 results in deficient extracellular matrix production [43], [45].

Reduced exposure of *Smad3^−/−^* mice to DSS allowed animals to survive the acute phase of disease and was associated with development of colon cancer at later time points. The propensity toward colon cancer development in *Smad3* ^−/−^ animals is likely attributable to many abnormalities. First, the repair issues in DSS-treated *Smad3* ^−/−^ mice unveiled at higher DSS concentrations may manifest as a ‘leaky repair’ at lower DSS concentrations resulting in chronic exposure to gut microflora and subsequent chronic inflammation. Second, immune cells in unmanipulated *Smad3^−/−^* mice are dysregulated and express increased levels of proinflammatory cytokines compared to WT mice [15], [16]. Last, the repair and immune abnormalities, in concert with elevated oncogene expression (*c-myc*) in *Smad3* ^−/−^ epithelial cells [16], may favor increased progression to cancer as compared to *Smad3* ^+/−^ and WT animals.

In contrast to the disease-promoting role of the dysregulated immune response in *Smad3^−/−^* mice, our data also demonstrate that adaptive immune cells play a protective, SMAD3-independent role in DSS-induced disease as *Smad3^−/−^* mice lacking T and B cells (*Smad3/Rag*-*DKO* mice) developed increased DSS-induced inflammation and cancer compared to T and B cell sufficient *Smad3^−/−^* mice. This result is the same as in our previous studies where *Helicobacter* was used to induce disease in *Smad3^−/−^* mice [17]. Although we did not compare disease in *Smad3/Rag-DKO* mice to *Rag2^−/−^* control mice in the studies reported here, we and others have investigated inflammation-associated cancer development in *Helicobacter*-infected *Rag2* ^−/−^ mice indicating that tumor development in these control mice are minimal in the time frames over which our studies were completed [17], [46], [47], [48]. Others utilizing *Rag-*deficient animals in inflammation-associated colon cancer studies [49], [50] administer both AOM and DSS, suggesting that factors in addition to inflammation are needed for robust tumor induction in *Rag*-deficient animals.

Minimal tumor formation in DSS-treated *Smad3^+/−^* compared to DSS-treated *Smad3^−/−^* mice suggests that the presence of at least one copy of *SMAD3* is sufficient to protect mice from development of dysplasia and invasive carcinoma. However, *Smad3^+/−^* mice did develop clinical signs of colitis, including bloody diarrhea and weight loss, but only a small number of animals developed severe disease requiring euthanasia. IBD scores at study end points were reduced compared with *Smad3^−/−^* mice and were similar to IBD scores in WT mice; tumor occurrence was rare in *Smad3^+/−^* mice.

DSS exposure induced a wide range of lesions in *Smad3^−/−^, Smad3^+/−^* and *Smad3/Rag-DKO* mice. We have used conservative criteria to classify invasive neoplasia using previously described morphological characteristics [6] which are similar to those used for human patients [5], [51]. Although the most common morphological type induced was mucinous adenocarcinoma (MAC), the there was a spectrum of proliferative lesions with variable gal-3 staining (**6**). Poorly differentiated invasive adenocarcinoma lacked gal-3 staining and well-differentiated mucinous adenocarcinomas had cytoplasmic gal-3 staining. Decreased galectin-3 signal in poorly differentiated regions of human tumors has been reported [28], [29]. The location of gal-3 signal is considered to be important prognostically in the characterization of human colorectal cancer where loss of nuclear localization is reported as part of the neoplastic progression [28]. However, Schoeppner (1995) and Endo (2005) noted increased gal-3 associated with high grade dysplasia and invasive tumors and distant lymph node metastasis [30], [31].

We recognize the controversy over the designation of neoplasia (especially MACs) in this and other mouse inflammation-driven colon cancer models due to the fact that the mouse colonic epithelium often has a florid reparative response that mimics dysplastic morphology [19], [52] and that frank metastatic disease has only rarely been reported [5], [15]. The reasons for the paucity of metastatic disease in the mouse model is likely multifactorial. The biology and humane use of mice may limit chances to induce and detect metastatic disease. Mice are frequently euthanized prior to development of macroscopic metastatic disease due to the impact of the primary tumor on the morbidity of the individual animal via secondary lesions such as intussusception, severe tumor associated inflammation, impaction or compression of abdominal organs. Surgical debulking of tumors to allow more time for development of metastatic disease is rarely done.

DSS-exposed *Smad3^−/−^* mice may be a useful animal model since they develop a spectrum of changes, some of which have similarities to human gastrointestinal lesions. The spectrum includes colitis with and without florid reparative hyperplasias (adenomatous hyperplasia), hyperplasia with herniation resembling colitis cystica profunda (CCP) [53], adenomas, disseminated peritoneal adenomucinosis similar to that described in humans as a subtype of pseudomyxomatous peritonei (PMP) [54], adenocarcinomas and mucinous adenocarcinomas. In humans, the differential diagnoses for space-occupying mucinous lesions of the colon and abdomen may include CCP, PMP and invasive mucinous colorectal adenocarcinoma (MAC). CCP is a result of inflammation coupled with herniation of mucosa into the submucosa. Mucin-filled retention cysts result in expansion of the submucosa and often rupture, mimicking invasive carcinoma [53]. PMP is a clinicopathologic syndrome of mucinous ascites and peritoneal lesions histologically characterized by mucin-producing well differentiated epithelium associated with pools of extracellular mucin and fibrosis forming expansile cysts. These are pathologically classified as disseminated peritoneal adenomucinosis (DPAM) and are thought to arise from ruptured low-grade mucinous tumors (adenomas) of the appendix [54]. Morbidity is a result of bowel obstruction secondary to mucus accumulation and fibrosis rather than metastatic spread. A more aggressive form of PMP, peritoneal mucinous carcinomatosis (PMCA), is diagnosed histologically due to the increased amount of mucinous epithelium with notable cytological atypia and complexity often with parenchymal organ involvement and lymph node metastasis [55]. It is possible that the lesions previously described in the various mouse models of inflammation-induced colon cancer also represent a morphological spectrum of lesions including CCP and PMP in addition to the reported carcinoma. An evolution of the consensus criteria for the classification and diagnosis of these controversial mucinous lesions in mouse models of inflammation-driven colorectal cancer and the validity of these designations in respect to the human conditions (CCP, PMP and invasive colorectal MAC) should be considered.

## Supporting Information

Figure S1 **Purified diet and ventilated caging worsens disease in** ***Smad3^−/−^*** **mice.** *Smad3^−/−^* mice were placed on either AIN93M purified diet or rodent chow 5053(Purina) 10 days prior to treatment with DSS. Because of rapid development of disease, animals receiving 2% and 3% DSS were necropsied at day 6. Animals given 1.5% DSS were necropsied at day 10. Weight loss (A) *Smad3^−/−^* mice receiving purified AIN93M diet lost significantly (student’s T test) more weight than*^−^* mice on rodent chow 5053. (B) IBD scores are significantly (Mann-Whitney) higher in *Smad3^−/−^* mice fed purified AIN93M diet. (C) Mice from two separate studies in two different caging systems (microisolator and ventilated) were given 1.5% DSS for 7 days. Survival was significantly decreased for mice housed in ventilated caging possibly due to increased water intake due to decreased humidity in ventilated cages compared to static caging.
(TIF)

Figure S2 **Histopathology scores of DSS-treated** ***Smad3^+^*** ^**/−**^ **and WT animals treated with varying doses of DSS.** *Smad3^+/−^* and WT mice were treated were treated with either a single DSS cycle or 9 cycles of DSS. A) IBD scores are shown for individual animals in each treatment group. Significant results of pair-wise comparisons (Mann-Whitney) of DSS-treated animals comparing WT vs. *Smad3^+/−^* genotypes as well as the different levels of DSS exposure among the same genotype are indicated. Summed dysplasia scores (B) were not significantly different from zero (Wilcoxon signed-rank test). *P≤0.05, **P≤*0.01*.
(TIF)

## References

[1] Bernstein CN, Blanchard JF, Kliewer E, Wajda A (2001) Cancer risk in patients with inflammatory bowel disease: a population-based study. Cancer 91: 854–862. 11241255 10.1002/1097-0142(20010215)91:4<854::aid-cncr1073>3.0.co;2-z

[2] Westbrook AM, Szakmary A, Schiestl RH (2010) Mechanisms of intestinal inflammation and development of associated cancers: lessons learned from mouse models. Mutation research 705: 40–59. 20298806 10.1016/j.mrrev.2010.03.001 PMC2878867

[3] Mladenova D, Kohonen-Corish MR (2012) Review: Mouse models of inflammatory bowel disease–insights into the mechanisms of inflammation-associated colorectal cancer. In vivo 26: 627–646. 22773577

[4] Kanneganti M, Mino-Kenudson M, Mizoguchi E (2011) Animal models of colitis-associated carcinogenesis. Journal of biomedicine & biotechnology 2011: 342637. 21274454 10.1155/2011/342637 PMC3025384

[5] Washington MK, Powell AE, Sullivan R, Sundberg JP, Wright N, et al (2013) Pathology of rodent models of intestinal cancer: progress report and recommendations. Gastroenterology 144: 705–717. 23415801 10.1053/j.gastro.2013.01.067 PMC3660997

[6] Boivin GP, Washington K, Yang K, Ward JM, Pretlow TP, et al (2003) Pathology of mouse models of intestinal cancer: consensus report and recommendations. Gastroenterology 124: 762–777. 12612914 10.1053/gast.2003.50094

[7] Bierie B, Moses HL (2010) Transforming growth factor beta (TGF-beta) and inflammation in cancer. Cytokine & growth factor reviews 21: 49–59. 20018551 10.1016/j.cytogfr.2009.11.008 PMC2834863

[8] Lampropoulos P, Zizi-Sermpetzoglou A, Rizos S, Kostakis A, Nikiteas N, et al (2012) TGF-beta signalling in colon carcinogenesis. Cancer letters 314: 1–7. 22018778 10.1016/j.canlet.2011.09.041

[9] Xavier RJ, Podolsky DK (2007) Unravelling the pathogenesis of inflammatory bowel disease. Nature 448: 427–434. 17653185 10.1038/nature06005

[10] Maloy KJ, Powrie F (2011) Intestinal homeostasis and its breakdown in inflammatory bowel disease. Nature 474: 298–306. 21677746 10.1038/nature10208

[11] Xu L (2006) Regulation of Smad activities. Biochimica et biophysica acta 1759: 503–513. 17182123 10.1016/j.bbaexp.2006.11.001 PMC1805629

[12] Munoz NM, Upton M, Rojas A, Washington MK, Lin L, et al (2006) Transforming growth factor beta receptor type II inactivation induces the malignant transformation of intestinal neoplasms initiated by Apc mutation. Cancer research 66: 9837–9844. 17047044 10.1158/0008-5472.CAN-06-0890

[13] Biswas S, Chytil A, Washington K, Romero-Gallo J, Gorska AE, et al (2004) Transforming growth factor beta receptor type II inactivation promotes the establishment and progression of colon cancer. Cancer research 64: 4687–4692. 15256431 10.1158/0008-5472.CAN-03-3255

[14] Takaku K, Oshima M, Miyoshi H, Matsui M, Seldin MF, et al (1998) Intestinal tumorigenesis in compound mutant mice of both Dpc4 (Smad4) and Apc genes. Cell 92: 645–656. 9506519 10.1016/s0092-8674(00)81132-0

[15] Zhu Y, Richardson JA, Parada LF, Graff JM (1998) Smad3 mutant mice develop metastatic colorectal cancer. Cell 94: 703–714. 9753318 10.1016/s0092-8674(00)81730-4

[16] Maggio-Price L, Treuting P, Zeng W, Tsang M, Bielefeldt-Ohmann H, et al (2006) Helicobacter infection is required for inflammation and colon cancer in SMAD3-deficient mice. Cancer Res 66: 828–838. 16424015 10.1158/0008-5472.CAN-05-2448 PMC5367923

[17] Maggio-Price L, Treuting P, Bielefeldt-Ohmann H, Seamons A, Drivdahl R, et al (2009) Bacterial infection of Smad3/Rag2 double-null mice with transforming growth factor-beta dysregulation as a model for studying inflammation-associated colon cancer. Am J Pathol 174: 317–329. 19119184 10.2353/ajpath.2009.080485 PMC2631344

[18] Deane NG, Manning HC, Foutch AC, Washington MK, Aronow BJ, et al (2007) Targeted imaging of colonic tumors in smad3−/− mice discriminates cancer and inflammation. Molecular cancer research : MCR 5: 341–349. 17426249 10.1158/1541-7786.MCR-06-0225

[19] Perse M, Cerar A (2012) Dextran sodium sulphate colitis mouse model: traps and tricks. Journal of biomedicine & biotechnology 2012: 718617. 22665990 10.1155/2012/718617 PMC3361365

[20] Lencioni KC, Drivdahl R, Seamons A, Treuting PM, Brabb T, et al (2011) Lack of effect of murine norovirus infection on a mouse model of bacteria-induced colon cancer. Comparative medicine 61: 219–226. 21819691 PMC3123754

[21] Burich A, Hershberg R, Waggie K, Zeng W, Brabb T, et al (2001) Helicobacter-induced inflammatory bowel disease in IL-10- and T cell-deficient mice. Am J Physiol Gastrointest Liver Physiol 281: G764–778. 11518689 10.1152/ajpgi.2001.281.3.G764

[22] Moolenbeek C, Ruitenberg EJ (1981) The “Swiss roll”: a simple technique for histological studies of the rodent intestine. Laboratory animals 15: 57–59. 7022018 10.1258/002367781780958577

[23] Fort MM, Mozaffarian A, Stover AG, Correia Jda S, Johnson DA, et al (2005) A synthetic TLR4 antagonist has anti-inflammatory effects in two murine models of inflammatory bowel disease. Journal of immunology 174: 6416–6423. 10.4049/jimmunol.174.10.6416 15879143

[24] Mahler M, Bristol IJ, Leiter EH, Workman AE, Birkenmeier EH, et al (1998) Differential susceptibility of inbred mouse strains to dextran sulfate sodium-induced colitis. The American journal of physiology 274: G544–551. 9530156 10.1152/ajpgi.1998.274.3.G544

[25] Okayasu I, Hatakeyama S, Yamada M, Ohkusa T, Inagaki Y, et al (1990) A novel method in the induction of reliable experimental acute and chronic ulcerative colitis in mice. Gastroenterology 98: 694–702. 1688816 10.1016/0016-5085(90)90290-h

[26] Cooper HS, Murthy SN, Shah RS, Sedergran DJ (1993) Clinicopathologic study of dextran sulfate sodium experimental murine colitis. Laboratory investigation; a journal of technical methods and pathology 69: 238–249. 8350599

[27] Tsuboi K, Shimura T, Masuda N, Ide M, Tsutsumi S, et al (2007) Galectin-3 expression in colorectal cancer: relation to invasion and metastasis. Anticancer research 27: 2289–2296. 17695516

[28] Lotz MM, Andrews CW Jr, Korzelius CA, Lee EC, Steele GD Jr, et al (1993) Decreased expression of Mac-2 (carbohydrate binding protein 35) and loss of its nuclear localization are associated with the neoplastic progression of colon carcinoma. Proceedings of the National Academy of Sciences of the United States of America 90: 3466–3470. 7682704 10.1073/pnas.90.8.3466 PMC46321

[29] Arfaoui-Toumi A, Kria-Ben Mahmoud L, Ben Hmida M, Khalfallah MT, Regaya-Mzabi S, et al (2010) Implication of the Galectin-3 in colorectal cancer development (about 325 Tunisian patients). Bulletin du cancer 97: E1–8. 20080461 10.1684/bdc.2010.1032

[30] Schoeppner HL, Raz A, Ho SB, Bresalier RS (1995) Expression of an endogenous galactose-binding lectin correlates with neoplastic progression in the colon. Cancer 75: 2818–2826. 7773932 10.1002/1097-0142(19950615)75:12<2818::aid-cncr2820751206>3.0.co;2-#

[31] Endo K, Kohnoe S, Tsujita E, Watanabe A, Nakashima H, et al (2005) Galectin-3 expression is a potent prognostic marker in colorectal cancer. Anticancer research 25: 3117–3121. 16080575

[32] Radosavljevic G, Volarevic V, Jovanovic I, Milovanovic M, Pejnovic N, et al (2012) The roles of Galectin-3 in autoimmunity and tumor progression. Immunologic research 52: 100–110. 22418727 10.1007/s12026-012-8286-6

[33] Hale LP, Cianciolo G (2008) Treatment of experimental colitis in mice with LMP-420, an inhibitor of TNF transcription. Journal of inflammation 5: 4. 18331642 10.1186/1476-9255-5-4 PMC2322983

[34] Loddenkemper C, Keller S, Hanski ML, Cao M, Jahreis G, et al (2006) Prevention of colitis-associated carcinogenesis in a mouse model by diet supplementation with ursodeoxycholic acid. International journal of cancer Journal international du cancer 118: 2750–2757. 16385573 10.1002/ijc.21729

[35] De Robertis M, Massi E, Poeta ML, Carotti S, Morini S, et al (2011) The AOM/DSS murine model for the study of colon carcinogenesis: From pathways to diagnosis and therapy studies. Journal of carcinogenesis 10: 9. 21483655 10.4103/1477-3163.78279 PMC3072657

[36] Clapper ML, Cooper HS, Chang WC (2007) Dextran sulfate sodium-induced colitis-associated neoplasia: a promising model for the development of chemopreventive interventions. Acta pharmacologica Sinica 28: 1450–1459. 17723178 10.1111/j.1745-7254.2007.00695.x

[37] Tanaka T, Kohno H, Suzuki R, Yamada Y, Sugie S, et al (2003) A novel inflammation-related mouse colon carcinogenesis model induced by azoxymethane and dextran sodium sulfate. Cancer science 94: 965–973. 14611673 10.1111/j.1349-7006.2003.tb01386.x PMC11160237

[38] Suzuki R, Kohno H, Sugie S, Tanaka T (2005) Dose-dependent promoting effect of dextran sodium sulfate on mouse colon carcinogenesis initiated with azoxymethane. Histology and histopathology 20: 483–492. 15736053 10.14670/HH-20.483

[39] Ni J, Chen SF, Hollander D (1996) Effects of dextran sulphate sodium on intestinal epithelial cells and intestinal lymphocytes. Gut 39: 234–241. 8991862 10.1136/gut.39.2.234 PMC1383305

[40] Dieleman LA, Palmen MJ, Akol H, Bloemena E, Pena AS, et al (1998) Chronic experimental colitis induced by dextran sulphate sodium (DSS) is characterized by Th1 and Th2 cytokines. Clinical and experimental immunology 114: 385–391. 9844047 10.1046/j.1365-2249.1998.00728.x PMC1905133

[41] Owen CR, Yuan L, Basson MD (2008) Smad3 knockout mice exhibit impaired intestinal mucosal healing. Laboratory investigation; a journal of technical methods and pathology 88: 1101–1109. 18711354 10.1038/labinvest.2008.77 PMC3971647

[42] Ashcroft GS, Yang X, Glick AB, Weinstein M, Letterio JL, et al (1999) Mice lacking Smad3 show accelerated wound healing and an impaired local inflammatory response. Nature cell biology 1: 260–266. 10559937 10.1038/12971

[43] Arany PR, Flanders KC, Kobayashi T, Kuo CK, Stuelten C, et al (2006) Smad3 deficiency alters key structural elements of the extracellular matrix and mechanotransduction of wound closure. Proceedings of the National Academy of Sciences of the United States of America 103: 9250–9255. 16754864 10.1073/pnas.0602473103 PMC1474013

[44] Tokumasa A, Katsuno T, Tanaga TS, Yokote K, Saito Y, et al (2004) Reduction of Smad3 accelerates re-epithelialization in a murine model of colitis. Biochemical and biophysical research communications 317: 377–383. 15063768 10.1016/j.bbrc.2004.03.047

[45] Latella G, Vetuschi A, Sferra R, Zanninelli G, D’Angelo A, et al (2009) Smad3 loss confers resistance to the development of trinitrobenzene sulfonic acid-induced colorectal fibrosis. European journal of clinical investigation 39: 145–156. 19200168 10.1111/j.1365-2362.2008.02076.x

[46] Erdman SE, Rao VP, Poutahidis T, Ihrig MM, Ge Z, et al (2003) CD4(+)CD25(+) regulatory lymphocytes require interleukin 10 to interrupt colon carcinogenesis in mice. Cancer research 63: 6042–6050. 14522933

[47] Boulard O, Kirchberger S, Royston DJ, Maloy KJ, Powrie FM (2012) Identification of a genetic locus controlling bacteria-driven colitis and associated cancer through effects on innate inflammation. The Journal of experimental medicine 209: 1309–1324. 22734048 10.1084/jem.20120239 PMC3405508

[48] Erdman SE, Poutahidis T, Tomczak M, Rogers AB, Cormier K, et al (2003) CD4+ CD25+ regulatory T lymphocytes inhibit microbially induced colon cancer in Rag2-deficient mice. The American journal of pathology 162: 691–702. 12547727 10.1016/S0002-9440(10)63863-1 PMC1851156

[49] Becker C, Fantini MC, Neurath MF (2006) TGF-beta as a T cell regulator in colitis and colon cancer. Cytokine & growth factor reviews 17: 97–106. 16298544 10.1016/j.cytogfr.2005.09.004

[50] Thaker AI, Rao MS, Bishnupuri KS, Kerr TA, Foster L, et al.. (2013) IDO1 Metabolites Activate beta-catenin Signaling to Promote Cancer Cell Proliferation and Colon Tumorigenesis in Mice. Gastroenterology 145: 416–425 e414. 10.1053/j.gastro.2013.05.002 PMC3722304 23669411

[51] Hamilton SR, Aaltonen LA (2000) Pathology and Genetics of Tumours of the Digestive System. World Health Organization Classification of Tumours Lyon: International Agency for Research on Cancer (IARC) Press.. 105–119.

[52] Percy DH, Barthold SW (2007) Pathology of Laboratory Rodents and Rabbits, Third Edition. Ames, Iowa: Blackwell Publishing. page 61 p.

[53] Molavi D, Argani P (2008) Distinguishing benign dissecting mucin (stromal mucin pools) from invasive mucinous carcinoma. Advances in anatomic pathology 15: 1–17. 18156808 10.1097/PAP.0b013e31815e52aa

[54] Ronnett BM (2003) Pseudomyxoma peritonei:pathologic features, site of origin and prognosis. CME Journal of Gynecologic Oncology 8: 192–198.

[55] Ronnett BM, Yan H, Kurman RJ, Shmookler BM, Wu L, et al (2001) Patients with pseudomyxoma peritonei associated with disseminated peritoneal adenomucinosis have a significantly more favorable prognosis than patients with peritoneal mucinous carcinomatosis. Cancer 92: 85–91. 11443613 10.1002/1097-0142(20010701)92:1<85::aid-cncr1295>3.0.co;2-r

