# Supplementary figures and images for: Characterization of Dextran Sodium Sulfate-Induced Inflammation and Colonic Tumorigenesis in Smad3 −/− Mice with Dysregulated TGFβ
Source: PLoS One. 2013 Nov 11;8(11):e79182. doi: 10.1371/journal.pone.0079182 (PMC3823566; doi:10.1371/journal.pone.0079182)

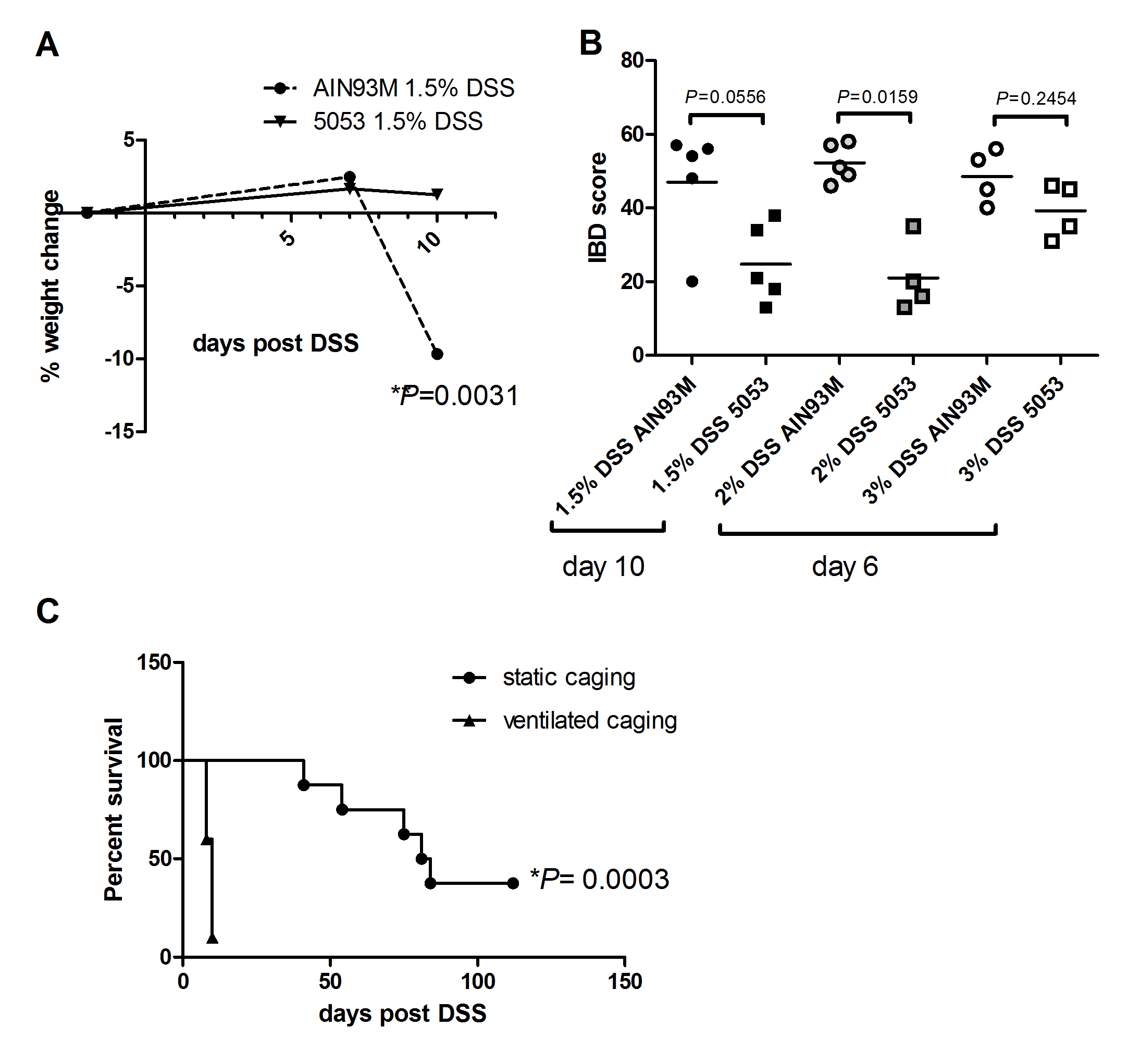

Supplement: Figure S1 — Purified diet and ventilated caging worsens disease in Smad3−/− mice. Smad3−/− mice were placed on either AIN93M purified diet or rodent chow 5053(Purina) 10 days prior to treatment with DSS. Because of rapid development of disease, animals receiving 2% and 3% DSS were necropsied at day 6. Animals given 1.5% DSS were necropsied at day 10. Weight loss (A) Smad3−/− mice receiving purified AIN93M diet lost significantly (student’s T test) more weight than− mice on rodent chow 5053. (B) IBD scores are significantly (Mann-Whitney) higher in Smad3−/− mice fed purified AIN93M diet. (C) Mice from two separate studies in two different caging systems (microisolator and ventilated) were given 1.5% DSS for 7 days. Survival was significantly decreased for mice housed in ventilated caging possibly due to increased water intake due to decreased humidity in ventilated cages compared to static caging. (TIF) [file pone.0079182.s001.tif]

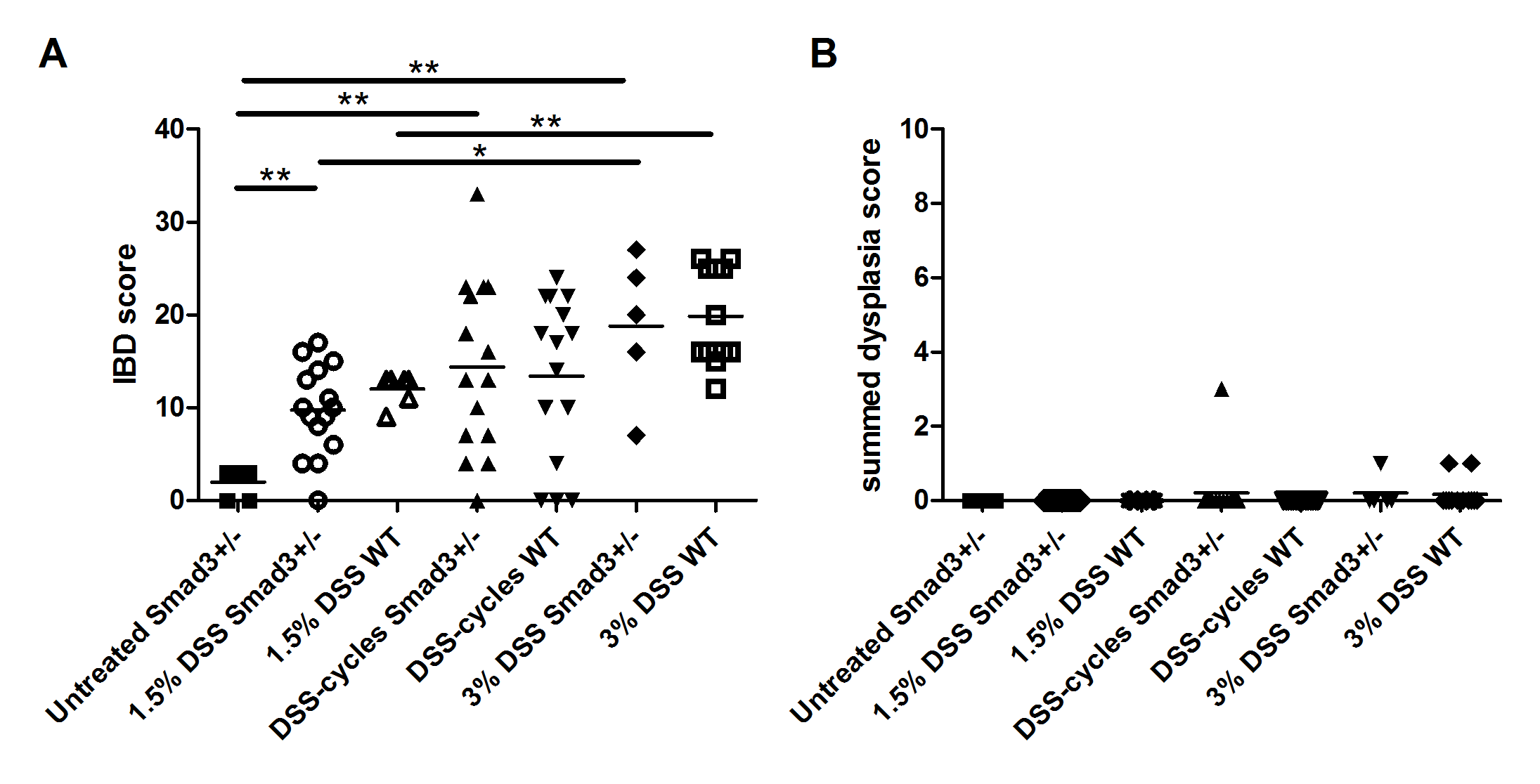

Supplement: Figure S2 — Histopathology scores of DSS-treated Smad3+ /− and WT animals treated with varying doses of DSS. Smad3+/− and WT mice were treated were treated with either a single DSS cycle or 9 cycles of DSS. A) IBD scores are shown for individual animals in each treatment group. Significant results of pair-wise comparisons (Mann-Whitney) of DSS-treated animals comparing WT vs. Smad3+/− genotypes as well as the different levels of DSS exposure among the same genotype are indicated. Summed dysplasia scores (B) were not significantly different from zero (Wilcoxon signed-rank test). *P≤0.05, **P≤0.01. (TIF) [file pone.0079182.s002.tif]
